# Supplementary material for: Evolution of secretin family GPCR members in the metazoa
Source: BMC Evol Biol. 2006 Dec 13;6:108. doi: 10.1186/1471-2148-6-108 (PMC1764030; doi:10.1186/1471-2148-6-108)
Supplement: Additional File 1 — List of the protostome (nematodes and arthropods) and tunicate (Ciona) putative family 2 GPCRs identified. The total size of each receptor protein sequence used in the in silico analysis performed is indicated within brackets and their sequences are available as additional data (Additional files 3, 4, 5, 6). The EST data available for each receptor and source of information are also indicated. No ESTs were available for the C. briggsae and mosquito receptor genes identified. The N-terminal region of CinS5A was identified by sequence comparison with the paralogue gene CinS5B and the C-terminal end of CinS93 was predicted using exon prediction programmes and sequence similarity approaches with the vertebrate homologue genes. [file 1471-2148-6-108-S1.pdf]

| *NEMATODES* | Receptor (size) | ESTs available | Source |
|---|---|---|---|
| *Caenorhabditis elegans (Cel)* | CelC18B12.2 (454 aa) | yk1433c05.3<br>AY314779<br>OSTR220F4_1<br>yk222b3.5<br>OSTR220F4_2<br>OSTF220F4_1<br>yk1192g04.5<br>yk648d8.5 | http://www.wormbase.org |
| | CelC13B9.4 (546 aa) | yk1596a10.3<br>AY314778<br>yk554f8.3<br>yk1101h12.3<br>yk1541e12.3<br>yk1284f12.3<br>yk1625f12.3<br>yk1404c05.3<br>yk1691c01.5<br>yk1116d06.3<br>yk1541e11.3<br>yk1523b07.3<br>yk1523b07.5<br>AY314776<br>yk1596a10.5<br>yk1101h12.5<br>OSTR053G1_1<br>cm20c7.5<br>yk1404c05.5<br>yk1625f12.5<br>yk1418b09.5<br>yk1284f12.5<br>AY314777 | http://www.wormbase.org |
| | CelZK643.3 (480 aa) | yk1615e01.5<br>yk1065b06.3<br>EB997588<br>EC021882<br>EC032209 | http://www.wormbase.org |
| *Caenorhabditis briggsae (Cbr)* | CbrCAE63268 (453 aa) | *Not available* | http://www.sanger.ac.uk |
| | CbrCAE70126 (643 aa) | *Not available* | http://www.sanger.ac.uk |
| | CbrCAE62707 (405 aa) | *Not available* | http://www.sanger.ac.uk |

| | | | |
|---|---|---|---|
| ***ARTHROPODS*** | | | |
| *Drosophila melanogaster (Dme)* | DmeCG13758 (585 aa) | RH51443<br>RH51348<br>RH30205<br>RH29191<br>RH55628<br>RH56268<br>RH56377<br>RH58612 | http://flybase.bio.indiana.edu |
| | DmeCG8422 (504 aa) | GH25339<br>GH15162<br>GH13988<br>GH13202<br>GH13988<br>GH10626<br>GH27289 | http://flybase.bio.indiana.edu |
| | DmeCG12370 (350 aa) | EK222913 | http://flybase.bio.indiana.edu |
| | DmeCG32843 (443 aa) | RE24343<br>RE13540<br>EK143812<br>EK155416<br>RE24343<br>EK016402<br>EP01810<br>EK170821<br>RH72515<br>RH71382 | http://flybase.bio.indiana.edu |
| | DmeCG4395 (561 aa) | EP20017<br>EK056607 | http://flybase.bio.indiana.edu |
| *Anopheles gambiae (Aga)* | AgaENSANGP00000014363 (348 aa) | *Not available* | http://www.ensembl.org |
| | AgaENSANGP00000014114 (201 aa) | *Not available* | http://www.ensembl.org |
| | AgaENSANGP00000020176 (427 aa) | *Not available* | http://www.ensembl.org |
| | AgaENSANGP00000014164 (465 aa) | *Not available* | http://www.ensembl.org |
| | AgaENSANGP00000004125 (427 aa) | *Not available* | http://www.ensembl.org |

***TUNICATE***

| | | | |
|---|---|---|---|
| *Ciona Intestinalis (Cin)* | CinS93 (413 aa) | cign020o11 | http://genome.jgi-psf.org |
| | CinS2303 (193 aa) | *Not available* | http://genome.jgi-psf.org |
| | CinS70 (374 aa) | *Not available* | http://genome.jgi-psf.org |
| | CinS5A (489 aa) | ciad086l11 | http://genome.jgi-psf.org |
| | CinS5B (476 aa) | cibd019i07 | http://genome.jgi-psf.org |
| | CinS273 (346 aa) | *Not available* | http://genome.jgi-psf.org |
| | CinS752 (465 aa) | ciad099i23 | http://genome.jgi-psf.org |
| | CinS372 (444 aa) | cinc021l09<br>cinc022e02<br>rcinc022e02<br>rcinc021l09<br>rcibd073e14<br>cibd073e14<br>rciad090c12<br>ciad090c12 | http://genome.jgi-psf.org |
| | CinS50 (398 aa) | cinc026m18<br>cinc031j15<br>rcinc026m18<br>cinc029i19<br>rcinc031j15 | http://genome.jgi-psf.org |
