# Supplementary material for: Evolution of secretin family GPCR members in the metazoa
Source: BMC Evol Biol. 2006 Dec 13;6:108. doi: 10.1186/1471-2148-6-108 (PMC1764030; doi:10.1186/1471-2148-6-108)
Supplement: Additional File 2 — Sequences of the protostome and deuterostome family 2 GPCRs TM domains used in phylogenetic analysis. Amino acid sequences of the human (Hsa), Takifugu (Tru), Ciona (Cin), Drosophila (Dme), Mosquito (Aga), C. elegans (Cel) and C. briggsae (Cbr) TM2, TM4, TM5 and TM6 domain regions used in the construction of the phylogenetic tree. [file 1471-2148-6-108-S2.pdf]

Receptor	*TM2*	*TM4*	*TM5*	*TM6*
HsaCGRPR	TLHKNLFFSFVCNSVVTIIHLTAVA	LMWYYFLGWGFPLIPACIHAIARSLY	YIIHGPICAALLVNLFFLLNIVRVLI	AVRATLILVPLLGIEFVLIPW
HsaCALR	TLHKNMFLTYILNSMIIIIHLVEVV	LRWYYLLGWGFPLVPTTIHAITRAVY	YIIHGPVMAALVVNFFFLLNIVRVLV	AVKATMILVPLLGIQFVVFPW
HsaCRFR1	IIHWNLISAFILRNATWFVVQLTMS	KWMFICIGWGVPFPIIVAWAIGKLYY	YIYQGPMILVLLINFIFLFNIVRILM	AVKATLVLLPLLGITYMLFFV
HsaCRFR2	VIHWNLITTFILRNVMWFLLQLVDH	KCLFLFIGWCIPFPIIVAWAIGKLYY	YIYQGPMILVLLINFIFLFNIVRILM	AVKATLVLLPLLGITYMLFFV
HsaGIPR	YIHINLFTSFMLRAAAILSRDRLLP	FRYYLLLGWGAPALFVIPWVIVRYLY	WIIRTPILMTILINFLIFIRILGILL	LARSTLTLVPLLGVHEVVFAP
HsaGLP1R	YIHLNLFASFILRALSVFIKDAALK	FRLYVSIGWGVPLLFVVPWGIVKYLY	LIIRLPILFAIGVNFLIFVRVICIVV	LAKSTLTLIPLLGTHEVIFAF
HsaGLP2R	YIHMNLFASFILRTLAVLVKDVVFY	WPRYLLLGWAFPVLFVVPWGFARAHL	WIIRGPMMLCVTVNFFIFLKILKLLI	LAKSTLVLIPLLGVHEILFSF
HsaGCGR	AIHANLFASFVLKASSVLVIDGLLR	FSLYLGIGWGAPMLFVVPWAVVKCLF	WILRFPVFLAILINFFIFVRIVQLLV	LAKSTLTLIPLLGVHEVVFAF
HsaPTR1	YIHMHLFLSFMLRAVSIFVKDAVLY	LWGFTVFGWGLPAVFVAVWVSVRATL	WIIQVPILASIVLNFILFINIVRVLA	LLKSTLVLMPLFGVHYIVFMA
HsaPTHR2	YIHMHLFVSFMLRATSIFVKDRVVH	LWGFILIGWGFPAAFVAAWAVARATL	WIYQAPILAAIGLNFILFLNTVRVLA	LAKSTLVLVLVFGVHYIVFVC
HsaPAC	FIHMNLFVSFMLRAISVFIKDWILY	FYWYTIIGWGTPTVCVTVWATLRLYF	WVIKGPVVGSIMVNFVLFIGIIVILV	LARSTLLLIPLFGIHYTVFAF
HsaVPAC1	YIHMHLFISFILRAAAVFIKDLALF	FWGYILIGWGVPSTFTMVWTIARIHF	WIIKGPILTSILVNFILFICIIRILL	LARSTLLLIPLFGVHYIMFAF
HsaVPAC2	YIHLNLFLSFILRAISVLVKDDVLY	FLAYLLIGWGLPTVCIGAWTAARLYL	WVIRIPILISIIVNFVLFISIIRILL	LAKSTLLLIPLFGVHYMVFAV
HsaGHRF	YVHTQLFTTFILKAGAVFLKDAALF	FWWLVLAGWGLPVLFTGTWVSCKLAF	WIIKGPIVLSVGVNFGLFLNIIRILV	LSKSTLFLIPLFGIHYIIFNF
HsaSCTR	YIHMHLFVSFILRALSNFIKDAVLF	LQGFVAFGWGSPAIFVALWAIARHFL	WIIRGPVILSILINFILFINILRILM	LAKSTLLLIPLFGIHYIVFAF
TruCRFR1	IIHWNLITAFILRNATWFIVQLTMS	KWMFICIGWGIPFPIIVAWAIGKLYY	YIYQGPMILVLVINFIFLFNIVRILM	AVKATLVLLPLLGITYMLFFV
TruCRFR2	IIHWNLITTFILRNVMWFLLQLIDH	KWVFLFIGWCIPCPIIVAWAIGKLYY	YIYQGPVILVLLINFVFLFNIVRILM	AVKATLVLLPLLGITYMLFFV
TruPTR1	YIHMHLFVSYMLRALSIFVKDVVLY	LWGFTLIGWGVPAMFVTVWATVRAVF	WIYQVPILVAVVVNFVLFLNIIRVLA	LLKSTLVLMPLFGVHYIIFNA
TruPTHR2	YIHMHLFLSFMLRAVSIFVKDRVVH	LWGFILIGWGVPAVFVAVWAIVRAAL	WIYQVPILMAIALNFILFVNIVRVLA	LAKSTLVLVLVFGIHYIIFVG
TruPTHR3	YIHIHIFTSFICRAVSIFVKDAVLY	LWALIIIGWGVPAVFVSIWSARASL	WIYQVPILAAIVVNFLLFINIVRVLA	LLKSTLVLMPLFGVHYMVFMA
TruPAC1A	FIHMNLFVSFILRAISVFIKDGVLY	FYWYTIVGWGTPTICVTVWAVLRLHF	WWVKGPVVASIMINFVLFGIIIILV	LARSTLLLIPLFGIHYTVFTF
TruPAC1B	FIHMNLFVSFMLRAISVFIKDSVLY	FYWYIIIGWGTPTLCVTIWAVLRLHF	WVIKGPVLASIMINFVLFVGIIIILV	LARSTLLLIPLFGIHYTVFAF
TruVPAC1A	YIHMHLFVSFILKAIAVFVKDVVLY	FSAYILIGWGGPTVFIAAWSVAKAYY	WIIKTPILASILINFILFICIIRILR	LAKSTLLLIPLFGINFIVFAF
TruVPAC1B	YIHIHLFVAFILKAVTVFIKDVVLY	FWGYILIGWGAPSVFISAWVLTKAYL	WIIKTPILGTILVNFILFIRIIRILR	LAKSTLLLIPLFGINYIIFAF
TruVPAC2A	YIHLNLFVSFMLRAVAVLAKDTLLF	LPVYMLIGWGIPEVFMVAWVICRVNL	RLINWPIMASVIINFIFFISIIRILV	LAKSTLLLIPLFGINYVVFVY
TruVPAC2B	YIHLNLFLSFILRAVAVLAKDDILF	FIVYLFIGWGIPAVFVFVWVMMRIYL	WVINGPIGFSIMVNFLLFVSIIRILV	LAKSTLLMIPLFGIHYVIFVT
TruGHRF1A	YIHVNLFSSFILRASAVFIKDTVLF	FWWYILIGWGLPSAVLVLWVLTRFIY	WIIKGPITVSLLVNILIFINVIRILV	LAKSTLFLIPLFGMHYTVFAF
TruGHRF1B	YIHINLFFSFILRASAVFIKDAVLF	FWWYIVIGWGLPTTIITVWILTRHFY	WIIRVPITASLLVNFLIFINVIRILV	LAKSTLLLIPLFGMHYMVFAL
TruS381	NIHMNLFASFILRALSVLIKDALMD	FKIYLCIGWGMPLLFLVPWVMAKYWY	WIIRSLILLAVVINFLIFIHIIKILV	LAKSTLTLIPLLGIHHMVTIF
TruS537	NIHMNLFASFILRAVSILVKDAFLT	FYIYMAIGWGAPLMFVVPWITVKYLY	WIIRSPILFAYLINFFIFIRIIKILM	LAKSTLTLIPLLGIHAILFTF
TruS611	TLHKNLFFSFVLNSVITIIWLTAVA	LTWYYLLGWGFPLIPASIHAVARSYY	YIIHGPICAALLVNLFFLLNIVRVLI	AVRATLILVPLLGIQFVLFPY
TruS1267	TLHKNLFCSYVLNSALTIIYLVAVV	LHWYYLLGWGFPLVPASIHAVARKKY	YAVHGPIVAALLVNLFFLLNIIRVLV	AVRATLILVPLLGIQFVIFPW
TruS2353	TLHKNLFLSFVLNSIVTVIWLTTVV	LLWYYLLGWGFPLMPTVLHSVARHSY	YIIHGPICAALVVNLFFLLNIVRVLI	AVRATLILIPLLGIQFVLLPY
TruS7267	YIHMNLFVSFILRAMAVILKEIIFY	LKRYMLLGWGTPVLFVTPWTVVKILH	WIIRGPITLTFVINFCIFIKILMLLL	LAKAKLVLIPLLGIHEVVFMV
TruS6614	YIHANLFLSLILRAVSVIIKDTMLE	HLPYICLGWGTPLLFVIPWVVMKLLK	WIIRLPILFASLINFLIFMKILKVIL	LAKATLTLIPLFGIHEIIFIF
TruS12367	SLHKNMFLSFIINSIVTIMWLSLSV	LFWYYVLGWGFPFVPAITYAVARGIF	YIIHGPIYAALIVNFFFLLNIVRVLI	AVRATLILILPLLGAQFILFPV
CinS5A	YIHMNLMLSFIVRYVAVMVKDKVIE	FPIFMAFGWGATWIPIGIWIAFRITF	WILRAPILISIAINFIIFINIIRMIV	LARSTLALIPLLGIHYIVFMG
CinS5B	YIHMNLMLSFIVRYVAVMVKDKVLE	FPIFMAFGWGAPWIPIGIWVRERITF	WILSAPILISIAINFIIFINIIRMIV	LARSTLALIPLLGIHYIIFMG
CinS50	IIHWNFVMSLIIRNVTWFLLFGVGF	WGRCVLLGWGVPIPIMMFWAILKAKF	YIYLVPIGIVLLINGFIFCNFVCILA	VSKAFVFYFSLLGLTYLLFMV
CinS70	YIHMNLMAAFMFRGLIWILHSAAFR	MKAFLVLGWGLPWLPVVAYFVPKSLD	WIVKVPILISLLINFIIFVNVVCVVG	LTKSTLSLIPLLGTQYLITAF
CinS93	TMHKHLFVSYILNALASVLWLHSHT	LLIYTGLGWGFPALSLLLYVVTRFVL	YIIHGPIAIALVINFIILMNLLRVLL	AIKATLVLIPLLGSQHILLTI
CinS273	IIHWNFVMSLMLRNVLWICLYLFMG	WRFYVTVGWGFPVLIMSAWAATKSVL	WIFKVPVLIALLINFVIMINVIRILV	TAKAALVLFPLLGLTYVLFII
CinS372	YIHMHIFASFILRAVVIFVKDRVLY	LWRFSVTGWGVPILFVVPWAIVRAKL	WIYNGPIVVNAVINFLLFLNIIRVLW	LAKSTLVLIPMFGVHAIVFIG
CinS752	YIHMHLFASFILRAVVIFVKDRVLY	LWRFSVTGWGVPILFVVPWAIVRAKF	WIYNGPIIVANVINFLLFLNIIRVLW	LAKSTLVLIPMFGVHAIVFIG
DmeCG8422	TIHANLFFTYIMSALFWILLLSVQI	FNIYASIGWGGPALFVVTWAVAKSLT	WIYQGPVCAVLIINLTFLLRIMWVLI	AAKALLVLIPLFGITYLVVLA
DmeCG13758	KIHKNLFVAMVLQVIIRLTLYLDQF	LKFFSRLGWCVPILMTTVWARCTVMY	WILEGPRLAVILLNFCFLVNIIRVLV	AVRAAIVLLPLLGITNLLHQL
DmeCG32843	TLHMNLFASFAANNSLWLVWYLLVM	VKWLIAFGWGSPAIVIFVYSMARGLG	NILMVPVCISMFLNLLFLCNIVRVVL	AFRATLLLVPLLGLQYILTPF
AgaP14164	TIHVNLFLTYIMSSSLWILILSLQI	FRKYAIIGWGGPLIFVGAWAIAKPFF	WIIQGPSCAVLVINLIFLLRIMWVLI	ASKALLVLIPLLGITYLIVIY
CelC13B9.4	LLHLHLMIAMLMVVILRLVLYIDLI	LLPYFIAGYGIPLVHTMLWLLVVLIK	WILDGPRMAELVINLFFICNVIRVLY	SVKAAMMLLPLLGVPNIMQTI
CelC18B12.2	SIHKNLATAFVFRFAVLAIWTIVQT	WSLYLACGWGVPFVVVTAWALVHQYI	WILAGTMGSALIMNLIFLLMIVVILV	TIKATLLLVPLLGISNIPLFY
Cbr63268	SIHKNLATAFVFRFAVLAIWTIVQS	WSLYLACGWGFPFVVVSAWTIVHQYK	WILTGTMGTAWIMNLIFLLMIVVILV	TIKATLLLVPLLGVSNIPLFY
Cbr70126	LLHLHLMIAMLMVVIIRLVLYIDLI	LLIYCIAGYGWPLLHTGFWLGVVLYK	WILDGPRMLQLVVNLLFICNVIRVLY	SVKAAMMLLPLLGVPNIMQTI
