# Supplementary material for: Evolution of secretin family GPCR members in the metazoa
Source: BMC Evol Biol. 2006 Dec 13;6:108. doi: 10.1186/1471-2148-6-108 (PMC1764030; doi:10.1186/1471-2148-6-108)
Supplement: Additional File 3 — Multiple sequence comparisons of the metazoan CALR/CGRPR. Multiple sequence alignment carried out with the protostome and deuterostome putative CALR/CGRPR protein sequences. The protostome, tunicate and Takifugu receptor sequences were manually edited having in consideration their sequence similarity, identification of splice sites consensus sequences (AG/GT) and the existence of EST data. Conserved cysteine residues are indicated by (•) and TM domains named. The N-terminal regions were annotated according to their level of conservation of the protostome and deuterostome receptors. The amino acid residues annotated with closed boxes have been previously identified in Figure 3 and the novel amino acid residues and protein motifs are annotated by open boxes. Incomplete sequences are due to gaps or low quality sequence data within receptor gene sequences. The Takifugu S012367 receptor sequence was not included since TM1 was not identified. The existence of putative intronic sequences was when possible investigated using the EST data available (Additional file 1). The start codon was chosen as the methionine in the correct frame of the first exon and the end of each receptor gene was chosen as the first stop codon in the correct frame of the last exon. [file 1471-2148-6-108-S3.pdf]

```
HsaCALR    : -------------------------------------------------------------------------MRFTFTSRCLALFLLLN :  17
HsaCGRPR   : --------------------------------------------------------------------------------MEKKCTLYFL :  10
S000611    : ------------------------------------------------------------------------------------------ :   -
S001267    : ------------------------------------------------------------------------------------------ :   -
S002353    : ------------------------------------------------------------------------------------------ :   -
CinS93     : ------------------------------------------------------------------------------------------ :   -
DmeCG13758 : MTLLSNILDCGGCISAQRFTRLLRQSGSSGPSPSAPTAGTFESKSMLEPTSSHSLATGRVPLLHDFDASTTESPGTYVLDGVARVAQLAL :  90
DmeCG32843 : -------------------------------------------------------------------------------MSDQIGNPNAT :  11
CelC13B9.4 : ------------------------------------------------------------------------MADATSPFNVSILDN-ST :  17
CelC18B12.2: ------------------------------------------------------------------------------------------ :   -
CbrCAE6326 : ------------------------------------------------------------------------------------------ :   -
CbrCAE7012 : ------------------------------------------------------------------------MADATSPFNVSILDNSTA :  18

HsaCALR    : HPTPILPAFSNQTYPTIEPKPFLYVVGRKKMMDAQYKCYDRMQQLP--AYQGEGPYCNRTWDGWLCWDDTPAGVLSYQFCPDYFPDFDPS : 105
HsaCGRPrR  : VLLPFFMILVTAELEESPEDSIQLGVTRNKIMTAQYECYQKIMQDPIQQAEG--VYCNRTWDGWLCWNDVAAGTESMQLCPDYFQDFDPS :  98
S000611    : -----------------------LGITRNKIVTAQFECYQKIMKDNRDSRGRGEPMCNRPWDGWLCWDDTKAGVISEQHCPDYFQDFDPS :  67
S001267    : ------------------------------------------------------SYCSRNWDGWLCWDDTQAGTVASQNCPDYFFNTDPT :  36
S002353    : -----------------------------------ANHQTNLNQNQIAKNQFSCLQCNMTWDGWLCWDQTAAGTTTEQSCPDYYHNFDPQ :  55
CinS93     : ----------------------MYNFEFTRQQFNCFVDNLNISRNSKSNEFVCSSFCGAIWDGYACWPPTQSSKEAVQNCPSYLPRFNTE :  68
DmeCG13758 : EPTVMDALPDSDTEQVLGNLNSSAPWNLTLASAAATNFENCSALFVNYTLPQTGLYCNWTWDTLLCWPPTPAGVLARMNCPGGFHGVDTR : 180
DmeCG32843 : FSGSGSGSGTNVASIAESVAESGPDFDALRAACETRLNASGQLAGSGGPGAEAGTHCAGTFDGWLCWPDTAVGTSAYELCPDFITGFDPA : 101
CelC13B9.4 : KLSEMVESGWNVLASTSVQAFNEAMDVLEESYPLCKKMLDHNNLFPERDPNDTRIWCNATYDTVLCWPPTPANSSVTLQCPHMKGLDPNK : 107
CelC18B12.2: -----------------------------MTSIIRAEYAACQELKKLENSSYNPGGCSVDFDKSLCWASAHIGQQMTRDCPFTFCTAIPG :  61
CbrCAE6326 : -----------------------------MTSIIQTEYEACEKLKAQ-NNSYTGSGCSVDFDRSLCWASARLGQQMTRDCPFKFCTSIPG :  60
CbrCAE7012 : KLSEMVDSGWNVLASTSVQAFNGAMDVLEESYPLCKKMLDHNKLFPERDPNDTRIWCNATYDTVLCWPPTPANSSVTLQCPHMKGLDPNK : 108

HsaCALR    : ------------------------------------------------------------------------------------------ :   -
HsaCGRPR   : ------------------------------------------------------------------------------------------ :   -
S000611    : G----------------------------------------------------------------------------------------- :  68
S001267    : ------------------------------------------------------------------------------------------ :   -
S002353    : ------------------------------------------------------------------------------------------ :   -
CinS93     : ------------------------------------------------------------------------------------------ :   -
DmeCG13758 : ------------------------------------------------------------------------------------------ :   -
DmeCG32843 : R----------------------------------------------------------------------------------------- : 102
CelC13B9.4 : ------------------------------------------------------------------------------------------ :   -
CelC18B12.2: ------------------------------------------------------------------------------------------ :   -
CbrCAE6326 : ------------------------------------------------------------------------------------------ :   -
CbrCAE7012 : NIVKRCDEMGRWAGKKPGHYENPWGWTNFTVCFKIDYEDVKFSSCVRLLIRCNVFCHVTPHKVPYSNHTTRHQTHFLIRAILDTYLSNQP : 198

                                                                                          TM1
HsaCALR    : -----------EKVTKYCDEKGVWFKHPENNR-----TWSNYTMCN-------------AFTPEKLKNAYVLYYLAIVGHSLSIFTLVIS : 166
HsaCGRPR   : -----------EKVTKICDQDGNWFRHPASNR-----TWTNYTQCN-------------VNTHEKVKTALNLFYLTIIGHGLSIASLLIS : 159
S000611    : -----------EMVTKVCTDGGYWFQHPESNR-----TWTNYTRCC-------------EHTHEGR-TAMNLFYLALIGHGLSLTSLFIS : 128
S001267    : -----------EKATKYCGEDGQWFRHPDTNR-----TWSNYTLCN-------------ENTKAKLKSAYILFYMAIVGHALSIASLLIS :  97
S002353    : -----------AVASKVCTENGEWGRHPESNR-----TWTNYENCI-------------ANSTNHQTAAMTHFYLVIIGHTLSLVSLLIS : 116
CinS93     : -----------EFAVRPCQANGEWFRYPTTQY-----QWTNYSRCQLQ---------------KGQEAVWAKYRLAISGYSVSMITLLVA : 127
DmeCG13758 : -----------KFAIRKCELDGRWGSRPNATEVNP-PGWTDYGPCYKPEIIRLMQQMGSKDFDAYIDIARRTRTLEIVGLCLSLFALIVS : 258
DmeCG32843 : ------------YAHKECGLDGEWFKHPLTNK-----TWSNYTTCVN---------------LEDLNWRHTVNLISEVGYGTSLLAILLS : 160
CelC13B9.4 : ------------NITKDCHVSGVWSGRNAGEMGPTLPGWTNFTMCYTDEVIYIMQNLNNESLTIAQEVARNARKLEFVGLGLSLVSLILA : 185
CelC18B12.2: ----CEEIKDRYMVSRNCNSMGVWQ---DSNYTMCIKVVEEYAQCLQG-----------FCRVCPDVLRDLVISVSLTLSFVSVILLVAA : 133
CbrCAE6326 : ----CEDIKEKYMVSRNCNNQGVWQ---DTNYTMCIKVAEEYSQCLSG-----------FCRVCPDFLRDMVISVSLTLSIVSVILLVAA : 132
CbrCAE7012 : TSTKLYTVTSTENITKDCHVSGVWSGRNAAEMGPTLPGWTNFTMCYTDEVIYIMQNLNNESLTIAQEVARNARKLEFVGLGLSLVSLILA : 288
                                             TM2                                             TM3

HsaCALR    : LGIFVFFRKLTTIFPLNWKYRKALSLGCQRVTLHKNMFLTYILNSMIIIIHLVEVVPNGE-----------LVRRDPVSCKILHFFHQYM : 245
HsaCGRPR   : LGIFFYFKS----------------LSCQRITLHKNLFFSFVCNSVVTIIHLTAVANNQA-----------LVATNPVSCKVSQFIHLYL : 222
S000611    : LGIFFHFN-------------------CQRITLHKNLFFSFVLNSVITIIWLTAVAKNQE-----------MVQRNPTSCKVSMFIHLYL : 188
S001267    : LAISSTSGDKSDLG--------WIFLSCQRITLHKNLFCSYVLNSALPIIYLVAVVNNPE-----------VVSRN-VGCKVLHFEHMYM : 167
S002353    : LTIFFYFKS----------------LSCQRITLHKNLFLSFVLNSIVTVIWLTTVVNKQA-----------HTHHYSAICK-LLMFHLYL : 178
CinS93     : LFIFFHFRS----------------LQCQRVTMHKHLFVSYILNALASVLWLHSHTIPGVS---------------QVYCKFLHAIHQYT : 186
DmeCG13758 : LLIFCTFRS----------------LRNNRTKIHKNLFVAMVLQVIIRLTLYLDQFRRGNKEAATN-TSLSVIENTPYLCEASYVLLEYA : 331
DmeCG32843 : LAILGYFKS----------------LKCARITLHMNLFASFAANNSLWLVWYLLVMPNSE-----------LLHQSPMRCVALHITLHYF : 223
CelC13B9.4 : ISIFSYFRR----------------LRVFRNLLHLHLMIAMLMVVILRLVLYIDLIFTGENGPHTNSAEGKTINTMPIVCEGMFFFLEYF : 259
CelC18B12.2: IVLFSIFDS----------------IQCRRLSIHKNLATAFVFRFAVLAIWTIVQTTNVFQDCTR--LTPLPLWDYEWICKAILWFVIYF : 205
CbrCAE6326 : IILFSVFDS----------------IQCRRLSIHKNLATAFVFRFAVLAIWTIVQSTNLFQDCTR--FHPLPIWDLEWICKTILWFVIYF : 204
CbrCAE7012 : ISIFSYFRR----------------LRVFRNLLHLHLMIAMLMVVIIRLVLYIDLIFTGDIGPHTNTAEGKTINTMPILCEGMFFFLEYF : 362
                                            TM4                                             TM5

HsaCALR    : MACNYFWMLCEGIYLHT-LIVVAVFTEKQRLRWYYLLGWGFPLVPTTIHAITRAVYFNDNCWLSVETHLL-----YIIHGPVMAALVVNF : 329
HsaCGRPR   : MGCNYFWMLCEGIYLHT-LIVVAVFAEKQHLMWYYFLGWGFPLIPACIHAIARSLYYNDNCWISSDTHLL-----YIIHGPICAALLVNL : 306
S000611    : FGCNYFWMLCEGIYLHT-LIVVAVFAEKQHLTWYYLLSWGFPLIPASIHAVARSYYYND---KNSKTSLL-----YIIHGPICAALLVNL : 269
S001267    : LGCNYFWMLCEGIYLHT-LIVVAVFAEEQHLHWYYLLGWGFPLVPASIHAVARKKYFDDKCWMSVETHLL-----YAVHGPIVAALLVNL : 251
S002353    : LSCNYFWMLCEGIYLHT-LIVVAVFAEKQHLLWYYLLGWGFPLMPTVLHSVARHSYYNDKCWVSSDTSLL-----YIIHGPICAALVVNL : 262
CinS93     : ETSNYFWMLCEGIYLHT-LVVVSVFREKQNLLIYTGLGWGFPALSLLLYVVTRFVLDDSSCWATPTTDAA-----YIIHGPIAIALVINF : 270
DmeCG13758 : RTAMFMWMFIEGLYLHN-MVTVAVFQGSFPLKFFSRLGWCVPILMTTVWARCTVMYMDTSLGECLWNYNLTP-YYWILEGPRLAVILLNF : 419
DmeCG32843 : LLSNYSWMLCEGFYLHT-VLVAAFISEKRLVKWLIAFGWGSPAIVIFVYSMARGLGGTPEDNRHCWMNQTNYQ--NILMVPVCISMFLNL : 310
CelC13B9.4 : KTVTFCWMFLEGIYLNN-QIVFGFFNSEPKLLPYFIAGYGIPLVHTMLWLLVVLIKKDFKVERCLGSYYLEP-EFWILDGPRMAELVINL : 347
CelC18B12.2: NVASVMWMLIEGAFLYSRFTVFAMRHSDAPWSLYLACGWGVPFVVVTAWALVHQYISSQQTNSFCWLPYAQGLHLWILAGTMGSALIMNL : 295
CbrCAE6326 : QVASVMWMLIEGAFLYSRFTVFAMRHSDAPWSLYLACGWGFPFVVVSAWTIVHQYKSSQLPNSFCWVPYAQGNHLWILTGTMGTAWIMNL : 294
CbrCAE7012 : KTVTFAWMFLEGVYLNN-QIAFGFFR-EPKLLIYCIAGYGWPLLHTGFWLGVVLYKKDFKVERCLGSYYLEP-EFWILDGPRMLQLVVNL : 449
                                           TM6                                          TM7

HsaCALR    : FFLLNIVRVLVTKMRETHEAE-----SHMYLKAVKATMILVPLLGIQFVVFPWR----PSNKMLGKIYDYVMHSLIHFQGFFVATIYCFC : 410
HsaCGRPR   : FFLLNIVRVLITKLKVTHQAE-----SNLYMKAVRATLILVPLLGIEFVLIPWR----PEGKIAEEVYDYIMHILMHFQGLLVSTIFCFF : 387
S000611    : FFLLNIVRVLITKLKVTHKAE-----SSLYMKAVRATLILDPLLGIQFVLFPYK----PDGQVSSEIYDYIMHIFMHYQGLLVATIFCFF : 350
S001267    : FFLLNIIRVLVTKLRDTHRAE-----SNMYMKAVRATLILVPLLGIQFVIFPWR----PENRIAGEVYEYIMHILMHYQGLLVATIFCFF : 332
S002353    : FFLLNIVRVLITKLRVTHQAE-----SSLYMRAVRATLILIPLLGIQFVLLPYK----PDDPRVFESYMYIMEILMHYQGFLVSTIFCFF : 343
CinS93     : IILMNLLRVLLSKLR-ANRNN-----IQRYIGAIKATLVLIPLLGSQHILLTIALY-IPN-ASVLRVLSYITNVLSSFQGFAVAIIFCFC : 352
DmeCG13758 : CFLVNIIRVLVMKLRQSQASD-----IEQTRKAVRAAIVLLPLLGITNLLHQLAPL---KTATNFAVWSYGTHFLTSFQGFFIALIYCFL : 501
DmeCG32843 : LFLCNIVRVVLLKLNAPASIQGSCGPSRTVLQAFRATLLLVPLLGLQYILTPFRPA---PKHPWENTYEIISAFTASFQGLCVAILFCFC : 397
CelC13B9.4 : FFICNVIRVLYSKVRESNNTS-----EAGLKKSVKAAMMLLPLLGVPNIMQTIPFAPTRDNIMVFAVWTYTASFTYMYQGLMVASIYCFT : 432
CelC18B12.2: IFLLMIVVILVQKLRTENSAE-----SKKIWRTIKATLLLVPLLGISNIPLFYE----PEHP--SSVYMLGSAILQHSQGIFIAVLYCFL : 374
CbrCAE6326 : IFLLMIVVILVQKLRTENSAE-----SKKIWRTIKATLLLVPLLGVSNIPLFYE----PEHP--SSVYMLGSAILQHSQGIFIAVLYCFL : 373
CbrCAE7012 : LFICNVIRVLYSKVRESNNTS-----EAGLKKSVKAAMMLLPLLGVPNIMQTIPFAPTRDNIMVFAVWTYTASFTYMYQGLMVASIYCFT : 534
```

```
HsaCALR    : NNEVQTTVKRQWAQFKIQWN---------QRWGRRPSNRSARAAAAAAEAGDIPIYICHQEPRNEPANNQGEESAEIIPLNIIEQESSA- : 490
HsaCGRPR   : NGEVQAILRRNWNQYKIQFG---------NSFS-NSEALRSASYTVSTISDGP-GYSHDCPSEHLNGKSIHDIENVLLKPENLYN----- : 461
S000611    : N--GEAVLRRHWNQHNIQFG---------SSVGNHSDAMRSASYPASSITEVQGCYSIDGHSEHMNGKGFHDTDTSMLKSDSPFA----- : 424
S001267    : NGEV-------------------------------------------------------------------------------------- : 336
S002353    : NGEVQSVLRRHWNQQRMQFT---------GTFA-SADFFRSASYVASSLTEVHRCYSTESHAEHLNG---GKSYSDIFRSESPFV----- : 415
CinS93     : HEEVLIPLLG------------------SQHILLTIALYIPNASVLRVLSYITNVLSSFQGFAVAIIFCFCHEEVGRIT----------- : 413
DmeCG13758 : NGEVRHQVTQLSICLKPSLFPTKIYIFAVLLKSLATQLSVRGHPEWAPKRASMYSGAYNTAPDTDAVQPAGDPSATGKRISPPN------ : 585
DmeCG32843 : NGEVIAQMKRKWRMMCFSNR------------------PRTNSYTATQVSFVRCGPPLPGEEKV-------------------------- : 443
CelC13B9.4 : NKEVNHVLKTFYARYRLLHKSQNELRRGSRSVASHYAAKNGTANASAPQTNNADEFGKLSPFPSRSKKGSDDSTTKLMKDAVMEEEKNAN : 522
CelC18B12.2: NSEIQGALKRQLSKVPFEFFKTRNRFETERTYVPEARNATKNGVPMEEMNKTKNIESGENTESQDQVSTGKQIYSVSTKS---------- : 454
CbrCAE6326 : NSEIQGALKRQLSKVPFEFFKSRNRFETERTYVPETRNATKNGVPMEELNGTKKIESENIESQEQANSSDKQIYSVPTKS---------- : 453
CbrCAE7012 : NKEVNHVLKAFYARYRLLHKSQNELRRGSRSVASHYAAKNGTSNTNAQQANNADENGKLSPFPSRSKKGSDDSTTKLMKDTVMEEEKNAN : 624


HsaCALR    : ------------------------- :   -
HsaCGRPR   : ------------------------- :   -
S000611    : ------------------------- :   -
S001267    : ------------------------- :   -
S002353    : ------------------------- :   -
CinS93     : ------------------------- :   -
DmeCG13758 : ------------------------- :   -
DmeCG32843 : ------------------------- :   -
CelC13B9.4 : NNGYGSAGEMTPLREGSNRSTKSP- : 546
CelC18B12.2: ------------------------- :   -
CbrCAE6326 : ------------------------- :   -
CbrCAE7012 : NNGYGSAGEMTPLREGYEE------ : 643
```
