# Supplementary material for: Evolution of secretin family GPCR members in the metazoa
Source: BMC Evol Biol. 2006 Dec 13;6:108. doi: 10.1186/1471-2148-6-108 (PMC1764030; doi:10.1186/1471-2148-6-108)
Supplement: Additional File 4 — Multiple sequence comparisons of the metazoan CRFR. Multiple sequence alignment carried out with the protostome and deuterostome putative CRFR protein sequences. The protostome, tunicate and Takifugu receptor sequences were manually edited having in consideration their sequence similarity, identification of splice sites consensus sequences (AG/GT) and the existence of EST data. Conserved cysteine residues are indicated by (•) and TM domains named. The N-terminal regions were annotated according to their level of conservation of the protostome and deuterostome receptors. The amino acid residues annotated with closed boxes have been previously identified in Figure 3 and the novel amino acid residues and protein motifs are annotated by open boxes. Incomplete sequences are due to gaps or low quality sequence data within receptor gene sequences. TruCRFR2, CinS273 and AgaP14164 have incomplete N-terminal ends and TruCRFR2 was also found to contain an incomplete intracellular loop 3. Despite the availability of EST data for N-terminal of CinS50 two potencial methionines were identified and for these reason the putative exon 1 sequence was not included. The existence of putative intronic sequences was when possible investigated using the EST data available (Additional file 1). The start codon was chosen as the methionine in the correct frame of the first exon and the end of each receptor gene was chosen as the first stop codon in the correct frame of the last exon. [file 1471-2148-6-108-S4.pdf]

```
HsaCRFR1  : ---------------------------------------MGGHPQLRLVKALLLLGLNPVSASLQDQHCESLSLASNISG :  41
HsaCRFR2  : -------------------------------------------MDAALLHSLLEANCSLALAEELLLDGWGPPLDPEGPY :  37
TruCRFR1  : --------------------MRRTRFSLLQVCALVSLLPPGSLANLTCEALLMLSGNVSLQSLAAAWNQSLMNATSTCSG :  60
TruCRFR2  : -------------------------------------------------------------------------------- :   -
CinS50    : -------------------------------------------NHLEKVADKLLALENELPRHIACQIFRDCRLEDQPAG :  37
CinS273   : -------------------------------------------------------------------------------- :   -
DmeCG8422 : ------------------------------MSDHNHIDSVNASGSDPLLDLHNLDGIGESVELQCLVQEHIEASTYGNDS :  50
AgaP14164 : KPTLLPRRLLHMANETMPTTPTAAAIAADDGSGGSPFGEGNVTGISVENPSLVEGLLALAMNASGAERCRLQQQAEELLQ :  80

HsaCRFR1  : LQCNASVDLIGTCWPRSPAGQLVVRPCPAFFYGVRYNTTNNGYRECLANGSWAARVNYSECQEILNEE--KKSKVHYHVA : 119
HsaCRFR2  : SYCNTTLDQIGTCWPRSAAGALVERPCPEYFNGVKYNTTRNAYRECLENGTWASKINYSQCEPILDDKQ-RKYDLHYRIA : 116
TruCRFR1  : LYCETSIDGIGTCWPRSAAGHMVSRPCPEMFYGVRYNTTNNVYRKCLANGTWALKGNYSMCKAILHEE--KKGKMHYQIA : 138
TruCRFR2  : ------------------------------------------YRECMDNGTWALKSNYSNCEPILEEK--RKYPVHYKVA :  36
CinS50    : GICQATVDSMGVCFTQTKPGRVAYSGCPTILNGINYIG--NSSKECYMNGTWAIKSNYDSCIHRLSQK--EICKYHFVSW : 113
CinS273   : -FCPSMIDGLGTCFHQSKSGQTASVSCLEELNGIPYNTSDTVTRKCLEGGRWENRSEYN-CRPILDEK--QPCEIHFANV :  76
DmeCG8422 : GHCLTQFDSI-LCWPRTARGTLAVLQCMDELQGIHYDSSKNATRFCHANGTWEKYTNYDACAHLPAPESVPEFEVIVELP : 129
AgaP14164 : DVACPSFFDMVSCWPRTPPGTLAVLPCFAELKGVQYDSSQNATRFCNVDGTWDNYTDYDRCEHLEQPPPLPSFEPEIELP : 160

                   TM1                                                           TM2
HsaCRFR1  : VIINYLGHCISLVALLVAFVLFLRLRPGCTHWGDQADGALEVGAPWSGAPFQVRRSIRCLRNIIHWNLISAFILRNATWF : 199
HsaCRFR2  : LVVNYLGHCVSVAALVAAFLLFLALR-----------------------------SIRCLRNVIHWNLITTFILRNVMWF : 167
TruCRFR1  : VIINFLGHCISMVALLVAFFLFLCLR-----------------------------SIRCLRNIIHWNLITAFILRNATWF : 189
TruCRFR2  : LIINYLGHCISVGALVVAFILFLCLR-----------------------------SIRCLRNIIHWNLITTFILRNVMWF :  87
CinS50    : TVLTIVGRSISLFALCIAFASFCILR------------------------------RRNLKMIIHWNFVMSLIIRNVTWF : 163
CinS273   : VKISMAGRGLSLFTLIVAFIIFCSIS----------------------------YRSGCLY-IIHWNFVMSLMLRNVLWI : 127
DmeCG8422 : TIIYYIGYTLSLVSLSLALIVFAYFK-----------------------------ELRCLRNTIHANLFFTYIMSALFWI : 180
AgaP14164 : TLIYFVGYSISLAALVLAVAVLVYFK-----------------------------DLRCLRNTIHVNLFLTYIMSSSLWI : 211

                                      TM3                                     TM4
HsaCRFR1  : VVQLTMSPEVHQSNVGWCRLVTAAYNYFHVTNFFWMFGEGCYLHTAIVLTYS-TDRLRKWMFICIGWGVPFPIIVAWAIG : 278
HsaCRFR2  : LLQLVDH-EVHESNEVWCRCITTIFNYFVVTNFFWMFVEGCYLHTAIVMTYS-TERLRKCLFLFIGWCIPFPIIVAWAIG : 245
TruCRFR1  : IVQLTMSPEVHESNVVWCRLVTASFNYFHSTNFFWMFGEGCYLHTAIVLTYS-TDKLRKWMFICIGWCIPFPIIVAWAIG : 268
TruCRFR2  : LLQLIDH-NIHESNEPWCRLITTIYNYFVVTNFFWMFVPGCYLHTAIVMTYS-TDKLRKWVFLFIGWCIPCPIIVAWAID : 165
CinS50    : LLFGVGFSGNVN--MVGCRVFAILFNYALIVTFSWMMVEGIMIHRKLESPFMSMADSFWGRCVLLGWGVPIPIMMFWAIL : 241
CinS273   : CLYLFMGFSNNENKTIICPIMVTVFNYGQTTSYCWMFLEGIYLHRYVAIQLGNDDKLSWRFYVTVGWGFPVLIMSAWAAT : 207
DmeCG8422 : LLLSVQISIRSG--VGSCIALITLFHFFTLTNFFWMLVEGLYLYMLVVKTFS-GDNLRFNIYASIGWGGPALFVVTWAVA : 257
AgaP14164 : LILSLQITVKLE--VAGCIFLVTLFHYFSTTNFFWMLVEGLYLYMLVVQTFS-GDTLRFRKYAIIGWGGPLIFVGAWAIA : 288

                                                  TM5
HsaCRFR1  : KLYYDNEK---------CWFGKRPGVYTDYIYQGPMILVLLINFIFLFNIVRILMTKLR--------ASTTSETIQYRKA : 341
HsaCRFR2  : KLYYENEQ---------CWFGKEPGDLVDYIYQGPIILVLLINFVFLFNIVRILMTKLR--------ASTTSETIQYRKA : 308
TruCRFR1  : KLYYDNEK---------CWFGKRAGVYTDYIYQGPMILVLVINFIFLFNIVRILMTKLR--------ASTTSETIQYRKA : 331
TruCRFR2  : KLYYENEQ---------CWFGKEPGKYMDYIYQGPVILVLLINFVFLFNIVRILM------------------------A : 199
CinS50    : KAKFENKD---------CWLN-HSPKQVDYIYLVPIGIVLLINGFIFCNFVCILA-KCRG---RKAAYKHRNEASTIRSV : 307
CinS273   : KSVLETGT---------CWLPGQSLSNADYIFKVPVLIALLINFVIMINVIRILVVKLCNPPARRPADGSSIESTHYFKT : 278
DmeCG8422 : KSLTVTYS---TPEKYEINCPWMQETHVDWIYQGPVCAVLIINLTFLLRIMWVLITKLR--------SANTVETRQYRKA : 326
AgaP14164 : KPFFGSVSNLEHPSKLEIECSWMRESHIDWIIQGPSCAVLVINLIFLLRIMWVLITKLR--------SANTVETRQYRKA : 360

                 TM6                           TM7
HsaCRFR1  : VKATLVLLPLLGITYMLFFVNPGEDEVSRVVFIYFNSFLESFQGFFVSVFYCFLNSEVRSAIRKRWHRWQDKHSIRARVA : 421
HsaCRFR2  : VKATLVLLPLLGITYMLFFVNPGEDDLSQIMFIYFNSFLQSFQGFFVSVFYCFFNGEVRSAVRKRWHRWQDHHSLRVPMA : 388
TruCRFR1  : VKATLVLLPLLGITYMLFFVNPGEDEISQVVFIYFNSFLESFQGFFVSVFYCFLNSEVRSAVRKRFHRWQEQHSIRARMT : 411
TruCRFR2  : VKATLVLLPLLGITYMLFFVNPGEDDIAQIVFIYFNSFLQSFQGFFVSVFYCFLNGEVRSAVRKRWHRWQDNHSLRMRVA : 279
CinS50    : SKAFVFYFSLLGLTYLIFMVNPSNSSTGEIIFIYTNVILESFQGFFICLFHCFCNTHMREAVLRRVQTLRAHGNFPCFGI : 387
CinS273   : AKAALVLFPLLGLTYVLFIISPGYGTTGETVFLYFNTVLDSFQGFFVCLVYYYAHHDVQVEVSKKLRR------------ : 346
DmeCG8422 : AKALLVLIPLFGITYLVVLAGPSESGLMGHMFAVLRAVLLSTQGFSVSLFYCFLNSEVRNALRHHISTWRDTRTIQLNQN : 406
AgaP14164 : SKALLVLIPLLGITYLIVIYGPVEG-VGSHIFAITRAILLSTQGFVVSLLYCFLNSEVRQTLRHHFYRWRDERNILSGKV : 439

HsaCRFR1  : RAMSIPTSPTRVSFHSIKQSTAV--------------------------------------------------------- : 444
HsaCRFR2  : RAMSIPTSPTRISFHSIKQTAAV--------------------------------------------------------- : 411
TruCRFR1  : QAMSIPTSPSRVSFHSIKQSTSL--------------------------------------------------------- : 434
TruCRFR2  : RAMSIPTSPTRISFHSIKQTTAV--------------------------------------------------------- : 302
CinS50    : GIGKSYYYYCI--------------------------------------------------------------------- : 398
CinS273   : -------------------------------------------------------------------------------- :   -
DmeCG8422 : RRYTTKSFSKGGGSPRAESMRPLTSYYGRGKRESCVSSATTTTLVGQHAPLSLHRGSNNALHTMPTLAANAMSSGSTLSV : 486
AgaP14164 : NNHHRRPTFSKDNSPRSRTESTRLVL------------------------------------------------------ : 465

HsaCRFR1  : ------------------ :   -
HsaCRFR2  : ------------------ :   -
TruCRFR1  : ------------------ :   -
TruCRFR2  : ------------------ :   -
CinS50    : ------------------ :   -
CinS273   : ------------------ :   -
DmeCG8422 : MPRAISPLMRQGLEENSV : 504
AgaP14164 : ------------------ :   -
```
