# Supplementary material for: Evolution of secretin family GPCR members in the metazoa
Source: BMC Evol Biol. 2006 Dec 13;6:108. doi: 10.1186/1471-2148-6-108 (PMC1764030; doi:10.1186/1471-2148-6-108)
Supplement: Additional File 5 — Multiple sequence comparisons of the metazoan PTR. Multiple sequence alignment carried out with the putative PTR deuterostome protein sequences. Tunicate and Takifugu receptor sequences were manually edited having in consideration their sequence similarity, identification of splice sites consensus sequences (AG/GT) and the existence of EST data. Conserved cysteine residues are indicated by (•) and TM domains named. The N-terminal regions were annotated according to their level of conservation. The amino acid residues annotated with closed boxes have been previously identified in Figure 3 and the novel amino acid residues and protein motifs are annotated by open boxes. Incomplete sequences are due to gaps or low quality sequence data within receptor gene sequences. The existence of putative intronic sequences was when possible investigated using the EST data available (Additional file 1). The start codon was chosen as the methionine in the correct frame of the first exon and the end of each receptor gene was chosen as the first stop codon in the correct frame of the last exon. [file 1471-2148-6-108-S5.pdf]

```
HsaPTR1  : ---------------MGTARIAPGLALLLCCPVLSSAYALVDADDVMTKEEQIFLLHRAQAQCEKRLKEVLQRPASIMESDKGWTSASTS :  75
HsaPTHR2 : ---------------MAGLGASLHVWGWLMLGSCLLARAQLDSDGTITIEEQIVLVLKAKVQCELNITAQLQ-EGEGN------------ :  62
TruPTR1  : ----------------------MPLHHLDFLLISLFSTWCLVCADDVLTKEEQIGLLFRAKRTCEGNIKAKHKVPDGFCS---------- :  58
TruPTHR2 : MKLIPARKMFQPGEGKGSMKPDMWIMVINFLVHWTLADTQIEVDGPLSAQEQMIILYDIKLQCQQNLSNTDPGFTDEP------------ :  78
TruPTHR3 : -------------------MSAWTTASLFLSQCVILVSALIDADDVITRDEQIYVLIGAHAKCERKIKAQIALVK--------------- :  56
CinS372  : ---------------------------------------MADDGITTLQEVMEVVLLEAQRNCKRDLEAGLPPKDK-------------- :  37
CinS752  : -------------------MKNLIDDVNNEVSKTGRMETMADDGITTVREMMEVLMEAQG-NCKRDLEAGLPPMDK-------------- :  56

HsaPTR1  : GKPRKDKASGKLYPESEEDKEAPTGSRYRGRPCLPEWDHILCWPLGAPGEVVAVPCPDYIYDFNHKG--HAYRRCDRNGSWELVPGHNRT : 163
HsaPTHR2 : --------------------------------CFPEWDGLICWPRGTVGKISAVPCPPYIYDFNHKG--VAFRHCNPNGTWDFMHSLNKT : 118
TruPTR1  : ----------------------------------PEWDGIVCWPEGPPGKLVSTACPEYIYDFDHKG--LAYRRCDFNGTWEQASAN-RT : 111
TruPTHR2 : --------------------------------CPPEWDGLICWPRGSPGLVTRVPCPSYIYDFNHRG--HAYRTCDINGSWAVLEDWNKT : 134
TruPTRR3 : -----------------------------AGDCIPEWDGIICWPQSRAGQLVSVLCPEYIYDFNHRG--WAYRQCDASGHWEQVHSVNRT : 115
CinS372  : ---------------------------------DGEWDKITCWPNSAPGRTVRLPCPEYIIDFDHTG--HALRHCSRDGRWAMVRDTNRT :  92
CinS752  : ---------------------------------DGEWDKITCWPNSAPGRTVRLPCPEYIIDFDHTGICHALRHCSRDGRWAMVRDTNRT : 113
                                                  TM1                            TM2
HsaPTR1  : WANYSECVKFLTNETR--EREVFDRLGMIYTVGYSVSLASLTVAVLILAYFRRLHCTRNYIHMHLFLSFMLRAVSIFVKDAVLYSGATLD : 251
HsaPTHR2 : WANYSDCLRFLQPDISIGKQEFFERLYVMYTVGYSISFGSLAVAILIIGYFRRLHCTRNYIHMHLFVSFMLRATSIFVKDRVVHAHIGVK : 208
TruPTR1  : WANYNECAQFLYHYNHSHEKEVFHRLYLIYTVGYSISLGSLIVAVLILGYFRRLHCTRNYIHMHLFVSYMLRALSIFVKDVVLYSGSTLE : 201
TruPTHR2 : WTNYSECWHFLHHNYEEERQHFFDRLYVIYTVGYAVSFSSLMVAILIMGFFRRLHCTRNYIHMHLFLSFMLRAVSIFVKDRVVHSSAGLQ : 224
TruPTHR3 : WANYTECTTYLNSNYKS-QEVVFKRLHLMYTVGYSVSLASLLVAVFILCYFKRLHCTRNYIHIHLFTSFICRAVSIFVKDAVLYTVSGYS : 204
CinS372  : FSDYGSCN----IPREEDVVEMIRRGGDLYTVGYSFSLVALVFAMIILAYFKRLHCTRNYIHMHLFASFILRAVVIFVKDRVLYYGAGIL : 178
CinS752  : FSDYSSCN----IPREEDVVEMIRRGGDLYTVGYSFSLVALVFAMIILAYFKRLHCTRNYIHMHLFASFILRAVVIFVKDRVLYYGAGIL : 199
                                                            TM3                             TM4
HsaPTR1  : EAERLTEEELRAIAQ--APPPPATAAAGYAGCRVAVTFFLYFLATNYYWILVEGLYLHSLIFMAFFSEKKYLWGFTVFGWGLPAVFVAVW : 339
HsaPTHR2 : ELESLIMQDDPQNSIEATSVD----KSQYIGCKIAVVMFIYFLATNYYWILVEGLYLHNLIFVAFFSDTKYLWGFILIGWGFPAAFVAAW : 294
TruPTR1  : NMERVTVEELKSITE--APP---ANKTQFIGCKVVVTLFMYFLATNYYWILVEGLYLHSLIFMTFFSDRKYLWGFTLIGWGVPAMFVTVW : 286
TruPTHR2 : DFDSALLDN--TKSISMSPTD----KSRYVGCKVTVVLFIYFLATNYFWILVEGLYLHSLIFMAFRTANKYLWGFILIGWGVPAVFVAVW : 308
TruPTHR3 : SASDSTAMTS-----------------HMAGCKVAVTFFLYFLATNHFWILVEGLYLHSLIFMAFLSDKNYLWALIIIGWGVPAVFVSIW : 277
CinS372  : DINTPDGEMTLEALKNRVDEIDADRSSYLVGCKLVMTLFHYFVATNYYWILVEALYLHSLIFVAFFSDKKYLWRFSVTGWGVPILFVVPW : 268
CinS752  : DINTPDGEMTLEALKNRVDEIDADRSSYLIGCKLVMTLFHYFVATNYYWILVEALYLHSLIFVAFFSDKKYLWRFSVTGWGVPILFVVPW : 289
                                                 TM5                                TM6
HsaPTR1  : VSVRATLANTGCWDLSSGNKKWIIQVPILASIVLNFILFINIVRVLATKLRETN-AGRCDTRQQYRKLLKSTLVLMPLFGVHYIVFMATP : 428
HsaPTHR2 : AVARATLADARCWELSAGDIKWIYQAPILAAIGLNFILFLNTVRVLATKIWETN-AVGHDTRKQYRKLAKSTLVLVLVFGVHYIVFVCLP : 383
TruPTR1  : ATVRAVFADTECWDLSAGNLKWIYQVPILVAVVVNFVLFLNIIRVLATKLRETN-AGRCDTRQQYRKLLKSTLVLMPLFGVHYIIFNAMP : 375
TruPTHR2 : AIVRAALADARCWELSAGNIKWIYQVPILMAIALNFILFVNIVRVLATKIRETN-AGRYDTRKQYRKLAKSTLVLVLVFGIHYIIFVGMP : 397
TruPTHR3 : VSARASLADTHCWDISAGNLKWIYQVPILAAIVVNFLLFINIVRVLASKLWETN-TGKLDPRQQYRKLLKSTLVLMPLFGVHYMVFMALP : 366
CinS372  : AIVRAKLDDTACWDIAVTEYKWIYNGPIVVANVINFLLFLNIIRVLWYKMRERGPIGKTDNRRQYKKLAKSTLVLIPMFGVHAIVFIGMP : 358
CinS752  : AIVRAKFEDTGCWDIAVTEYKWIYNGPIIVANVINFLLFLNIIRVLWYKMRERGPIGKTDNRRQYKKLAKSTLVLIPMFGVHAIVFIGMP : 379
                                TM7
HsaPTR1  : YTEVSGTLWQVQMHYEMLFNSFQGFFVAIIYCFCNGEVQAEIKKSWSRWTLALDFKRKARSGSSSYS----YGPMVSHTSVTNVGPRVGL : 514
HsaPTHR2 : HSFTG-LGWEIRMHCELFFNSFQGFFVSIIYCYCNGEVQAEVKKMWSRWNLSVDWKRTPPCGSRRCG--------SVLTTVTHSTSSQSQ : 464
TruPTR1  : YTEVSGIPWVIQVHYEMLVKPLQGFLVAIIYCFCNGEVQAEIKKSWSRRTLALDFKRKARSGSNTYS----YGPMVSHTSVTNVTARAPL : 461
TruPTHR2 : HTFEG-LSWEVRMYCELFFNSFQGFFVSIIYCYCNGEVQTEIKKTWTRWNLNFNWDVPMVCGRYHYG--------SVVVGINNNNSTSSQ : 478
TruPTHR3 : YTEVTGLLWQVQMHYEMFFNSSQGFFVAFIYCFCNGEVQTEVKKAWLRRSLTQDIKQKSRMTSSAGGGSCYYGGMMSHTTMHSVSLSVVT : 456
CinS372  : DDISSGTWWDIRMSFDLFFNSFQGFFVAIIYCFCNGEVQAEFRKAWERFNLSVEIKRGRRERS-----------RSSVTMLTSFNSSASQ : 437
CiSn752  : DDISSGTWWDIRMSFDLFFNSFQGFFVAIIYCFCNGEVQAEFRKAWERFNLSVEIKRGRRERS-----------RSSVTMLTSFNSSASQ : 458

HsaPTR1  : GLPLSPR------------LLPTATTNGHPQLPGHAKPGTPALETLETTPPAMAAPKDDGFLNGSCSGLDEEASGPERPPALLQEEWETV : 592
HsaPTHR2 : ----VAASTRMVLISGKAAKIASRQPDSHITLPGYVWSNSEQDCLPHSFHEETKEDSGRQGDDILMEKPSRPMESNPDTEGCQGETEDVL : 550
TruPTR1  : ALHLTTR------------LGPVPVN-GHRNLPGYVKNGSVSENSVPSSGQELHVPDEE-------HPAPARPCEDQKPSSVVEEERETV : 531
TruPTHR2 : SHLAAGAPANSLVSSRVSRTTGPSMVSTHIILPGYVISNSDP------SIPEEPEDSGPKMVDDISLKENTPVLSPGATAEDEEETL--- : 559
TruPTHR3 : PRSLSVGGVVGSGGSTGGGSGLRPPRHNSLYPQSSLSTYVAADLETPQTQQELTLQRESESFKRHVRASKGAPHSRSPEASPVQSLREES : 546
CinS372  : QVRIMTS----------------------------------------------------------------------------------- : 444
CinS752  : QVRIMTS----------------------------------------------------------------------------------- : 465

HsaPTR1  : M------------ : 593
HsaPTHR2 : ------------- :   -
TruPTR1  : M------------ : 532
TruPTHR2 : ------------- :   -
TruPTHR3 : GNILSLKELETVL : 559
CinS372  : ------------- :   -
CinS752  : ------------- :   -
```
