# Supplementary material for: Evolution of secretin family GPCR members in the metazoa
Source: BMC Evol Biol. 2006 Dec 13;6:108. doi: 10.1186/1471-2148-6-108 (PMC1764030; doi:10.1186/1471-2148-6-108)
Supplement: Additional File 6 — Multiple sequence comparisons of the metazoan CGCR/GLPR/GIPR. Multiple sequence alignment carried out with the deuterostome putative CGCR/GLPR/GIPR protein sequences. Tunicate and Takifugu sequences were manually edited having in consideration their sequence similarity, identification of splice sites consensus sequences (AG/GT) and the existence of EST data. Conserved cysteine residues are indicated by (•) and TM domains named. The N-terminal regions were annotated according to their level of conservation. The amino acid residues annotated with closed boxes have been previously identified in Figure 3 and the novel amino acid residues and protein motifs are annotated by open boxes. Incomplete sequences are due to gaps or low quality sequence data within receptor gene sequences. Takifugu S006614 and S007267 and CinS70 have incomplete N-terminal regions and the Takifugu receptor S000381 was not included in the alignment since TM3 is frameshifted. The existence of putative intronic sequences was when possible investigated using the EST data available (Additional file 1). The start codon was chosen as the methionine in the correct frame of the first exon and the end of each receptor gene was chosen as the first stop codon in the correct frame of the last exon. The Ciona CinS5A, CinS5B and CinS70 were only compared with the vertebrate GCGR/GLPR/GIPR with which they share higher sequence similarity [file 1471-2148-6-108-S6.pdf]

```
HsaGCGR : --------------------------------------MPPCQPQRPLLLLLLLLACQPQVPSAQVMDFLFEKWKLYGDQCHHNLS-- :  48
HsaGLP1R: ------------------------------------MAGAPGPLRLALLLLGMVGRAGPRPQGATVSLWETVQKWREYRRQCQRSLTE- :  52
HsaGLP2R: MKLGSSRAGPGRGSAGLLPGVHELPMGIPAPWGTSPLSFHRKCSLWAPGRPFLTLVLLVSIKQVTGSLLEETTRKWAQYKQACLRDLL-- :  88
HsaGIPR : -------------------------------------MTTSPILQLLLRLSLCGLLLQRAETGSKGQTAGELYQRWERYRRECQETLA-- :  51
S000537 : -------------------------------------MHYGDYVLKTVTVRLQFS---------SANSLELVKEQWSSYKNQCLDYLS-- :  42
S006614 : ----------------------------------------------------------------------------QYKDDCIQMIMNE :  13
S007267 : ------------------------------------------------------------------------------------------ :   -
CinS5B  : -----------------------------------------------------------------------MCKYMKYKEQCEKSLNET :  18
CinS5A  : -----------------------------------------------------------------------MCKYMQYRERCEKSLNET :  18
CinS70  : ------------------------------------------------------------------------------------------ :   -

HsaGCGR : LLPPPTELVCNRTFDKYSCWPDTPANTTANISCPWYLPWHHK---VQHRFVFKRCGPDGQWVRGP-RGQPWRDASQCQMDG--EEIEVQK : 132
HsaGLP1R: DPPPATDLFCNRTFDEYACWPDGEPGSFVNVSCPWYLPWASS---VPQGHVYRFCTAEGLWLQKDNSSLPWRDLSECEESK----RGERS : 135
HsaGLP2R: --KEPSGIFCNGTFDQYVCWPHSSPG-NVSVPCPSYLPWWSE---ESSGRAYRHCLAQGTWQTIENATDIWQDDSECSENH---SFKQNV : 169
HsaGIPR : AAEPPSGLACNGSFDMYVCWDYAAPNATARASCPWYLPWHHH---VAAGFVLRQCGSDGQWG-------LWRDHTQCENP---EKNEAFL : 128
S000537 : SAEPSTGLVCNRTFDLYACWPDGLPGTTVNVSCPWFLPWYRK-----------------------------------------------A :  85
S006614 : PLSPGGGLFCNRTFDRYACWPDAPAGSLVNISCPFYLPWYEK---VSQGVVRRCGSDGLWERDD-SGHVWRDKSQCEEEEECHGLSLQL :  99
S007267 : ------GIYCKGTFDMFVCWPHSAPG-NVSVPCPSYLPWISE---DGSRRAHRECLENGTWRERENSSEPWRDDSECKEEH---YFKDKE :  77
CinS5B  : LKNFENQTFCPGFFDRQICWPHTPAGQLSTEPCPSYIEWR-----NESLVAYRFCEFDGSWMVRNDTGQSVQWVNRIQCDVATNGLQYEL : 103
CinS5A  : LKNFENQTFCPGIFDGYICWPHTPAGQLLTEPCPSYIEWKNVIHLQESLVAYRFCEFDGSWMVRNDTGQSVH-LDHLRGDHRTKMYVGNY : 107
CinS70  : ---------------------------------------------IPAMHCNATLGGWNERSSLHTCFNRSSDMDDDVASISN---D :  39

                                    TM1                                     TM2
HsaGCGR : EVAKMYSSFQVMYTVGYSLSLGALLLALAILGGLSKLHCTRNAIHANLFASFVLKASSVLVIDGLLRTRYSQKIGDDLSVSTWLSDGAVA : 222
HsaGLP1R: SPEEQLLFLYIIYTVGYALSFSALVIASAILLGFRHLHCTRNYIHLNLFASFILRALSVFIKDAALKWMY-STAAQQHQWDGLLSYQDSL : 224
HsaGLP2R: DRYALLSTLQLMYTVGYSFSLISLFLALTLLLFLRKLHCTRNYIHMNLFASFILRTLAVLVKDVVFYNSYSKRPDNENGWMSYLS-EMST : 258
HsaGIPR : DQRLILERLQVMYTVGYSLSLATLLLALLILSLFRRLHCTRNYIHINLFTSFMLRAAAILSRDRLLP-RPGPYLG-DQALALWN--QALA : 214
S000537 : HYGLILGQLRIMYTVGYSLSLGALLLALGILLAFRKLHCMRNNIHMNLFASFILRAVSILVKDAFLTLTLDSRSSNNTQAQAPVNTTGIT : 175
S006614 : WFKQMMVNFRMVYTVGYSISLLTLSTALVILLSFRKLRCSRNYIHANLFLSLILRAVSVIIKDTMLERHWGREIV--------KQTDAAI : 181
S007267 : DEMHLQTALRLISIIGYSLSLFSLTVATLVMAMLRKLHCTRNYIHMNLFVSFILRAMAVILKEIIFYIKHFNLPKDDPQWKSYADSAIVL : 167
CinS5B  : GPLEGGPKHNDIYTAGYIFSLVCMVIALFILMFFKKLHCTRNYIHMNLMLSFIVRYVAVMVKDKVLEDHYAVGQTNLTQMEMSQYCDDVS : 193
CinS5A  : LLLSIIDIVTKIYTAGYIFSLVCMVIALFILMFFKKLHCTRNYIHMNLMLSFIVRYVAVMVKDKVIEDHYAVGRPNLTQMEMSQYCDDVA : 197
CinS70  : DYNARYEILTRYVTVGHALSLATLLVALALLACLKNIHCTRIYIHMNLMAAFMFRGLIWILHSAAFRGEQDLMVEILNNLVTLQN----- : 124

                                   TM3                                        TM4
HsaGCGR : G-------CRVAAVFMQYGIVANYCWLLVEGLYLHNLLGLATLPERSFFSLYLGIGWGAPMLFVVPWAVVKCLF--ENVQCWTSNDNMGF : 303
HsaGLP1R: S-------CRLVFLLMQYCVAANYYWLLVEGVYLYTLLAFSVFSEQWIFRLYVSIGWGVPLLFVVPWGIVKYLY--EDEGCWTRNSNMNY : 305
HsaGLP2R: S-------CRSVQVLLHYFVGANYLWLLVEGLYLHTLLEPTVLPERRLWPRYLLLGWAFPVLFVVPWGFARAHL--ENTGCWTTNGNKKI : 339
HsaGIPR : A-------CRTAQIVTQYCVGANYTWLLVEGVYLHSLLVLVGGSEEGHFRYYLLLGWGAPALFVIPWVIVRYLY--ENTQCWERNEVKAI : 295
S000537 : W-------CRGAMVMMQYSVMANNYWLLVEGIYLHSLLVITVFSEKKYFYIYMAIGWGAPLMFVVPWTTVKYLY--ENEECWERNINMGF : 256
S006614 : G-------CRMAQAVMQYCVLANHCWFFGEAVYLYSVLIASVFIDNNKHLPYICLGWGTPLLFVIPWVVMKLLK--ENKECWAFNENMNY : 262
S007267 : S-------CRVSAVCMQYFVACNYFWLLVEAVFLHTLLFSAVLTKRRLLKRYMLLGWGTPVLFVTPWTVVKILH--ENTGCWSIMN-KWI : 247
CinS5B  : --------CRLVITLMHYAIIANYFWLLVEGVYLQ-LLLVFVMTEYKYFPIFMAFGWGAPWIPIGIWVAFRITF--ENVGCWEIYNALRT : 272
CinS5A  : GTDGLMVSCRLVITLMHYATIANYFWLLAEGVYLQ-LLLVFVMTEYKYFPIFMAFGWGATWIPIGIWIAFRITF--ENVGCWEVNNMIPI : 284
CinS70  : -------WCRLYNVFQIYFVTANYFWLLVEGLYLRLLLRAVLFSHTKYMKAFLVLGWGLPWLPVVAYFVPKSLDPEENRDCWYEDRNSAY : 207

                  TM5                                                 TM6
HsaGCGR : WWILRFPVFLAILINFFIFVRIVQLLVAKLRA-----RQMHHTDYKFRLAKSTLTLIPLLGVHEVVFAFVTDEHAQ--GTLRSAKLFFDL : 386
HsaGLP1R: WLIIRLPILFAIGVNFLIFVRVICIVVSKLKA-----NLMCKTDIKCRLAKSTLTLIPLLGTHEVIFAFVMDEHAR--GTLRFIKLFTEL : 388
HsaGLP2R: WWIIRGPMMLCVTVNFFIFLKILKLLISKLKA-----HQMCFRDYKYRLAKSTLVLIPLLGVHEILFSFITDDQVE--GFAKLIRLFIQL : 422
HsaGIPR : WWIIRTPILMTILINFLIFIRILGILLSKLRT-----RQMRCRDYRLRLARSTLTLVPLLGVHEVVFAPVTEEQAR--GALRFAKLGFEI : 378
S000537 : WWIIRSPILFAYLINFFIFIRIIKILMSKLRA-----HQMRYTDYKFRLAKSTLTLIPLLGIHAILFTFVIDESVQKGSLLRLIRLFYDL : 341
S006614 : WWIIRLPILFASLINFLIFMKILKVILSKLRAN----KQSGYPDYKLRLAKATLTLIPLFGIHEIIFIFATDEQTT--GILRYIKVFFTL : 346
S007267 : WWIIRGPITLTFVINFCIFIKILMLLLSKLKA-----DQLKFTDYRYRLAKAKLVLIPLLGIHEVVFMVLTDECME--GRSLYAKNFVNL : 330
CinS5B  : WWILSAPILISIAINFIIFINIIRMIVSKLRA-----NNMTRSDYKYRLARSTLALIPLLGIHYIIFMGVSDSVTN--STITKTKFAFEL : 355
CinS5A  : WWILRAPILISIAINFIIFINIIRMIVSKLSA-----NNMTRSDYKYRLARSTLALIPLLGIHYIVFMGVSDSVTDN-SAFINTKFAFEI : 368
CinS70  : WWIVKVPILISLLINFIIFVNVVCVVGAKLRVNQRQQADDRREDYKWRLTKSTLSLIPLLGTQYLITAFVHFDSEEN-PTAEFVKKAFEV : 296

             TM7
HsaGCGR : FLSSFQGLLVAVLYCFLNKEVQSELRRRWHRWRLGKVLWEERNTSN------------------------------------------- : 432
HsaGLP1R: SFTSFQGLMVAILYCFVNNEVQLEFRKSWERWRLEHLHIQRDSSMK------------------------------------------- : 434
HsaGLP2R: TLSSFHGFLVALQYGFANGEVKAELRKYWVRFLLARHSGCRACVLGKDFRFLGKCP--KKLSEGDGAEKLRKLQPSLNSGRLLHLAMRG- : 509
HsaGIPR : FLSSFQGFLVSVLYCFINKEVQSEIRRGWHHCRLRRSLGEEQRQLP------------------------------------------- : 424
S000537 : LFSSFQ----------------------------------------------------------------------------------- : 347
S006614 : TLNSFQGLLVSVLYCYANKEVRTELRRKWHIWRTEAEFV-------------------------------------------------- : 385
S007267 : TLNSFQGFLVAVLYCFANGEVQAELKKRWQLFLFTNHFQVRGCFQGKPLKQLWKCTQGRRPQCSRQSDSCDEGEASTTHPQLLQVAVHRG : 420
CinS5B  : ILTSLQGSIIAILYCFLNGEVQTEIRKCWRNWRWSHRLPAASGKFSTYRSDVQMTSITYAHSIDKGD---------------------- : 422
CinS5A  : ILTSLQGSIIAILYCFLNGEVQTEIRKCWRNWRWSHRLPAASGKFSTYRSDVQMTSITYAHSMDKGD---------------------- : 435
CinS70  : SFVSIQGLLVAFIYCFFNGEVQEECGKTWRRWKTRWELERRERKHHR------------------------------------------ : 343

HsaGCGR : ------HRASSSPGHGPPSKELQFGRGGGSQDSSAETPLAGG-LPRLAESPF----------------------------- : 477
HsaGLP1R: ------PLKCPTSSLSSGATAGSSMYTATCQASCS---------------------------------------------- : 463
HsaGLP2R: ------LGELGAQPQQ--DHARWPRGSSLSECS-EGDVTMANTMEEILEESEI---------------------------- : 553
HsaGIPR : ------ERAFRALPSGSGPGEVPTSRG----LSSGTLPGPGNEASRELESYC----------------------------- : 466
S000537 : ---------------------------------------------------------------------------------- :   -
S006614 : ---------------------------------------------------------------------------------- :   -
S007267 : GRAVGVRGEVKGQGSGGCDSAGLDLLTRKSLSSSDGELTLGETMGEILEESEF---------------------------- : 473
CinS5B  : ------AHETSLLSPNNKHDHVDNKRSSTASCVSDQQTKIGDSSRLMCNGNGSPSKQLVV--------------------- : 476
CinS5A  : ------AHETSLLSPNNKHDHVDNKRSSTASCVSDQQTKIGDSSRLMCNGNGSPSKQLVV--------------------- : 489
CinS70  : ------MQRRRSMNHSSITNLFTSVVTRKSSLYTGES-------------------------------------------- : 374
```
